# ADAPTING THE CAPABLE PROGRAM TO ADDRESS FUNCTION AND SOCIAL CONNECTION NEEDS AMONG HOMEBOUND OLDER ADULTS

**DOI:** 10.1093/geroni/igae098.0587

**Published:** 2024-12-31

**Authors:** Tiffany Riser, Wonkyung Jung, Samantha Curriero, Kennedy McDaniel, Thomas Cudjoe

**Affiliations:** Johns Hopkins University, Baltimore, Maryland, United States; Boston College, Baltimore, Maryland, United States; Johns Hopkins University, Baltimore, Maryland, United States; Johns Hopkins University, Baltimore, Maryland, United States; Johns Hopkins University School of Medicine, Baltimore, Maryland, United States

## Abstract

CAPABLE Care + Connect is a high-fidelity prototype based on the CAPABLE program that is currently being developed and implemented using a two-phase approach. The purpose of CAPABLE Care + Connect is to leverage human-centered design to adapt an evidence-based intervention (Phase 1) and to test the feasibility of implementing the tailored intervention in a home-based primary care setting (Phase 2). The overarching goal of CAPABLE Care + Connect is to better address the needs of homebound older adults experiencing limited life space and limited social connection. During the symposium presentation, we will share the methodological activities involved in developing this intervention using human-centered design. Additionally, we will present early insights gained from older adults, care partners, clinicians, and thought leaders regarding opportunities and challenges of integrating the CAPABLE and Johns Hopkins Home-Based Medicine programs. These insights will highlight the program’s role in facilitating aging in place while also supporting social connection in older adults with disabilities who are experiencing or at risk for social isolation. After attending this symposium session, participants will demonstrate an understanding of how human-centered design approaches can be applied to adapt an evidence-based intervention in a home-care setting. Additionally, participants will be able to summarize perspectives on social isolation, loneliness, and unmet needs for social connection in homebound older adults with disabilities.

